# Men treated with BEACOPP for Hodgkin lymphoma may be at increased risk of testosterone deficiency

**DOI:** 10.1007/s00277-023-05512-y

**Published:** 2023-10-23

**Authors:** Signe Micas Pedersen, Claus Larsen Feltoft, Torsten Holm Nielsen, Peter de Nully Brown, Anne Ortved Gang, Lars Møller Pedersen, Niels Jørgensen

**Affiliations:** 1 Department of Hematology, Copenhagen University Hospital—Rigshospitalet, Blegdamsvej 9, 2100 KBH Ø, Copenhagen, Denmark; 2 Danish Medicines Agency, Axel Heides Gade 1, 2300 KBH S, Copenhagen, Denmark; 3 Department of Clinical Medicine, University of Copenhagen, Blegdamsvej 3B, 2200 KBH N Copenhagen, Denmark; 4 Department of Hematology, Zealand University Hospital, Vestermarksvej 15, 4000 Roskilde, Denmark; 5 Department of Growth and Reproduction, Copenhagen University Hospital—Rigshospitalet, Blegdamsvej 9, 2100 KBH Ø, Copenhagen, Denmark; 6 Department of Endocrinology, Copenhagen University Hospital—Herlev and Gentofte, Borgmester Ib Juuls Vej 1, 2730 Herlev, Denmark

**Keywords:** Lymphoma, Sexual health, Testosterone, SHBG, LH, Free testosterone

## Abstract

In the current study, we report the prevalence of male testosterone deficiency in a cohort of 60 male long-term survivors of malignant lymphoma with normal total testosterone but in the lower part of the reference level. Testosterone deficiency was defined as subnormal concentrations of total testosterone or subnormal concentrations of calculated free testosterone. The aim was to clarify whether total testosterone was sufficient for identification of testosterone deficiency in male survivors of malignant lymphoma. Hormonal analyses taken at follow-up were compared with samples taken at diagnosis for a subgroup of 20 survivors, for evaluation of changes in hormones over time. Another group of 83 similar survivors of malignant lymphoma with testosterone in the high end of reference levels were also used for comparison, to identify groups of increased risk of testosterone deficiency. A total group of 143 survivors were therefore included in the study. Our findings indicate that for screening purposes an initial total testosterone is sufficient in some survivors because sexual hormone binding globulin concentration was found stable over time. However, 15% were found with subnormal calculated free testosterone. Survivors intensely treated for Hodgkin lymphoma and older survivors were identified as high-risk groups for testosterone deficiency necessitating endocrinological attention during follow-up. Some evidence of pituitary downregulation was also found, because of uncompensated decreases in testosterone concentration over time. In conclusion, longitudinal measurements of total testosterone alone do not seem adequate for the screening of testosterone deficiency for all long-term lymphoma survivors.

**Supplementary Information:**

The online version contains supplementary material available at 10.1007/s00277-023-05512-y.

## Introduction

Male malignant lymphoma survivors treated with chemotherapy have a higher prevalence of sexual dysfunction than men from the general population [1, 2]. The dysfunction is at least partly caused by a reduced serum concentration of testosterone [1, 3]. Calculated free testosterone (cFT) may be considered the biologically active testosterone and therefore the golden standard for measuring testosterone deficiency [4]. Nevertheless, most studies of sexual dysfunction use serum total testosterone (TT) for screening purposes, whereas only few include sexual hormone binding globulin (SHBG) with subsequent calculations of FT (systematic review submitted by this research group). Sexual dysfunction caused by testosterone deficiency may only be apparent when calculated free testosterone (cFT) is accounted for and thus, testosterone deficiency (TD) may be underestimated if the assessment is solely based on TT [5].

Serum concentrations of SHBG are susceptible to changes in the body and are shown to increase with age [5, 6] but decrease in obese [5, 7] and type 2 diabetic men [8]. However, altered liver function can also result in altered concentrations of SHBG, including increased levels, which may lead to lower cFT if TT does not increase accordingly [9]. Lymphoma patients are often treated with chemotherapy with potential impact on liver function, and some studies [10–14] but not all [15–17] have detected a trend toward increased SHBG concentrations among treated patients. However, most of these studies have evaluated the effects of treatments that are not in accordance with current intensified standard regimens. If such intensive therapies impact SHBG concentration, this may lead to reduced cFT in case the Leydig cells also loose capacity for testosterone production. Thus, patients may have reduced concentrations of cFT despite a normal concentration of TT.

Our aim with this study was to evaluate whether serum TT is sufficient for screening malignant lymphoma survivors with sexual dysfunction, or if the cFT improves diagnostic accuracy. Secondly, we aimed at investigating differently treated subgroups to identify if some of these might be at increased risk of testosterone deficiency. Thirdly, analytically immunoassays (IA) are frequently used in routine diagnostics of testosterone deficiency, and we therefore also aimed to elucidate if IA can be used interchangeably with the gold standard liquid chromatography with mass spectrometry (LC–MS/MS) [4], for the assessment of TD in male survivors of malignant lymphoma.

## Study population and methods

### Study population

**Fig. 1:**
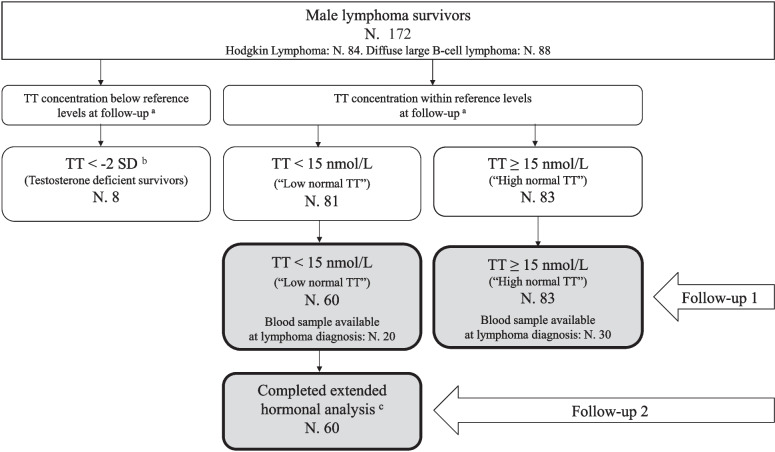
Flowchart of included male long-term survivors of malignant lymphoma. Gray box: the primary samples for this article. TT, total testosterone. ^a^Total testosterone alone was measured in a cross-sectional study (VitalityObs), and results are used as follow-up 1 samples. ^b^ − 2 SD = age adjusted reference levels: lower cutoff for age 18–49 years = 8.6 nmol/l, for age 50 years and above = 6.7 nmol/l. ^c^Total testosterone, calculated free testosterone, sexual hormone binding globulin, and luteinizing hormone were measured for 60 survivors included in a subsequent study (VitalityCheck) and represent follow-up 2. Blood samples available at diagnosis = samples collected from medical journal including total testosterone, calculated free testosterone, sexual hormone binding globulin, and luteinizing hormone

We included data from a cohort of adult male survivors of Hodgkin lymphoma (HL) and diffuse large B-cell lymphoma (DLBCL) diagnosed and treated with first-line treatment in the period 2008 to 2018 and in complete remission at inclusion. Survivors were identified through the Danish Lymphoma Registry [18]. The cohort consisted of 172 male survivors and has been described in a previous article [3]. As outlined in Fig. 1, eight patients with TT below the lower reference limit and additional 21 patients not available for blood samples at FU2 were not included in our data analysis (no contact: 11 survivors, declined inclusion because of lack of time: 3, did not complete the additional blood samples: 3; Online resource). Therefore, 143 survivors were included in this data analysis. The included cohort was divided into 83 survivors with TT of at least 15 nmol/l at FU1 (defined as “High normal TT”) and 60 survivors with TT < 15 nmol/l but above lower age adjusted reference limit at FU1 (defined as “Low normal TT”). Median time from diagnosis to follow-up was 7 years (range 2–13) and 8 years (range 4–14), respectively. Reference levels for age 18–49 years were 8.6 nmol/l and for age > 49 years 6.7 nmol/l (Fig. 1).

Clinical factors, including date of diagnosis, lymphoma diagnosis, treatment, and follow-up, were obtained from the Danish Lymphoma Registry. Information on sociodemographic factors, e.g., relationship status, sexual activity, erectile function, education, employment, children status, BMI, smoking, alcohol consumption, exercise, and comorbidity, was obtained by telephone interview or during a clinical visit.

### Hormones

At FU1, all 172 participants had a fasting venous blood sample drawn before 10 am. Samples were analyzed at the department of clinical biochemistry at Copenhagen University Hospital—Rigshospitalet, Denmark. Analyses were performed on a Cobas e801 analytical unit, with Elecsys Testosterone II assays from Roche. These results were used to stratify the men into one of three groups: eight men with TT below lower reference level (− 2 SD), 81 men with TT above − 2 SD but < 15 nmol/l (“Low normal TT”), and 83 men with TT ≥ 15 nmol/l (“High normal TT”) (Fig. 1).

The 81 men with “Low normal TT” were eligible for an extended hormonal determination, and 60 men accepted referral to the Department of Growth and Reproduction (Dept. of GR), Copenhagen University Hospital—Rigshospitalet, Denmark, for an extended hormonal determination, FU2. Twenty-one men were not included in these additional examinations as stated above. The extended investigation included renewed venous blood sampling in the morning hours determined for TT, SHBG, and LH. TT and SHBG were measured by chemiluminescence immunoassays using the Access 2 instrument (Beckman Coulter, Brea, CA, USA). The limits of detection (LODs) were 0.35 nmol/l for T and 0.33 nmol/l for SHBG, and the corresponding coefficients of variation (CVs) were below 4% and 8%, respectively. Total testosterone was also assessed by liquid chromatography–tandem mass spectrometry (LC–MS/MS). Assessment of serum concentrations of LH chemiluminescence immunoassays was performed using the Atellica instrument (Siemens Healthineers, Tarrytown, NY, USA) with LOD of 0.07 IU/l and inter-assay CV < 15%. All analyses were accredited by The Danish Accreditation Fund for medical examination according to a European and international standard approved in Denmark (the standard DS/EN ISO 15189). Free testosterone was calculated based on TT assessments and SHBG concentrations, using the formula proposed by Vermeulen et al. assuming a fixed albumin concentration of 43.8 g/l [4, 19].

Among the 143 men included in the current study, 20 men in the “Low normal TT” and 30 men in the “High normal TT” subgroups also had hormonal assessment performed at the time of diagnosis of lymphoma as part of semen cryopreservation prior to onset of chemotherapy. Venous blood samples had been taken during daytime from 8 am to 1 pm and assessed at Dept. of GR, and TT also assessed by use of immunoassay.

### Statistics

Statistical analyses were performed using the statistical software R, version R-4.1.2. Medians and range were used for descriptive statistics because of small subgroups, where both means and medians with standard deviations and percentiles were used for hormonal analyses. For evaluation of differences between paired samples, hormonal analyses were transformed using the natural logarithm to calculate changes in percent. Fisher’s exact test was used for comparison of groups for categorical variables to calculate *p* values, and paired *t*-test was used for continuous variables to calculate confidence intervals for paired samples, and unpaired *t*-tests for unpaired samples.

For evaluation of comparability of analyses-methods, we used linear regression analyses and Bland–Altman plots.

## Results

Among the 143 men included, 67 (47%) had been treated for HL, of whom 38 men (56%) were in stage I/II, and 76 (53%) had been treated for DLBCL, of whom 45 men (59%) were in stage I/II, at the time of diagnosis. Table 1 shows the sociodemographic and clinical features of the men stratified according to group: “High normal TT” or “Low normal TT” and diagnosis. HL survivors were younger when diagnosed than DLBCL survivors in both groups (“High normal TT”: *p* < 0.002, “Low normal TT”: *p* = 0.04). HL survivors with “Low normal TT” were not significantly older than HL survivors with “High normal TT” (*p* = 0.13), and no age difference was seen for DLBCL survivors (*p* = 0.63). Most men had received standard first-line treatment with either R-CHOP, ABVD, or BEACOPP according to lymphoma type. Distribution of treatment intensity and chemotherapy regimens received did not differ between the “High normal TT” and “Low normal TT” groups (*p* = 0.80 and *p* = 0.38). The number of men in a committed relationship (*p* = 0.68), offspring status (*p* = 0.74), education (*p* = 0.14), employment status (*p* = 1.0), hours of exercise per week (*p* = 0.28), number of standard drinks per week (*p* = 0.81), number of pack years (*p* = 0.23), and prevalence of ED (*p* = 0.24) were approximately equally distributed within the two groups. Survivors with “Low normal TT” had higher comorbidity scores (*p* = 0.03) and higher body mass indexes (BMI) (*p* < 0.002) compared to survivors with “High normal TT.”

**Table 1:** Clinical and epidemiological baseline characteristics of adult male lymphoma survivors according to testosterone at first follow-up and diagnosis [3]

	Survivors with “High normal TT” (no extended hormonal analysis) *N* 83			Survivors with “Low normal TT” (extended hormonal assessment) *N* 60		
	All *N* 83	HL *N* 39	DLBCL *N* 44	All *N* 60	HL *N* 28	DLBCL *N* 32
Age at inclusion, median (range)	48 (24–65)	36 (24–60)	55 (28–65)	47 (27–67)	44 (27–67)	54 (29–66)
Age at diagnosis, median (range)	41 (19–60)	28 (19–53)	48 (19–60)	40 (19–61)	37 (19–61)	44 (20–59)
Follow-up time from diagnosis to inclusion in years, median (range)	7.0 (2.0–13.0)	7.0 (2.0–13.0)	6.5 (3.0–12.0)	8.0 (4.0–14.0)	7.0 (4.0–14.0)	8.0 (4.0–14.0)
Ann Arbor stage, *N* (%)						
I/II	51 (61.5)	23 (59.0)	28 (63.6)	32 (53.3)	15 (53.6)	17 (53.1)
III/IV	32 (38.5)	16 (41.0)	16 (36.4)	28 (46.7)	13 (46.4)	15 (46.9)
Treatment, *N* (%)^a^						
ABVD	31 (37.4)	31 (79.5)	0	18 (30.0)	18 (64.3)	0
BEACOPP	8 (9.6)	8 (20.5)	0	10 (16.7)	10 (35.7)	0
R-CHOP	44 (52.0)	0	44 (100.0)	31 (51.6)	0	31 (96.9)
R-CODOX-M/R-IVAC	0 (0.0)	0	0	1 (1.6)	0	1 (3.1)
*N* of treatment cycles, median (range)^a^						
ABVD	–	4 (2–8)	–	–	5 (2–8)	–
BEACOPP	–	8 (6–8)	–	–	6 (6–8)	–
R-CHOP-like regimen	–	–	6 (3–8)	–	–	6 (3–8)
Married/committed relationship, *N* (%)	70 (84.3)	34 (87.2)	36 (81.8)	51 (85.0)	24 (85.7)	27 (83.4)
Offspring status, *N* (%)^b^						
Child conceived before treatment	41 (49.4)	11 (28.2)	30 (68.2)	34 (56.7)	9 (32.1)	25 (78.1)
Child conceived after treatment	15 (18.7)	10 (25.6)	5 (11.4)	11 (18.3)	9 (32.1)	2 (6.3)
Childless, unwanted	7 (8.4)	5 (12.8)	2 (4.6)	5 (8.3)	2 (7.1)	3 (9.4)
Childless, by choice	20 (24.1)	13 (33.3)	7 (15.9)	10 (16.7)	8 (28.6)	2 (6.3)
Highest level of education, *N* of years after primary school (%)						
0–3	11 (13.3)	5 (12.8)	6 (13.6)	8 (13.3)	4 (14.3)	4 (12.5)
4–5	24 (28.9)	7 (18.0)	17 (38.6)	28 (46.7)	12 (42.9)	16 (50.0)
6	17 (20.5)	10 (25.6)	7 (15.9)	10 (16.7)	5 (17.9)	5 (15.6)
8–9	31 (37.4)	17 (43.6)	14 (34.1)	14 (23.3)	7 (25.0)	7 (21.9)
Currently employed, *N* (%)	75 (90.4)	37 (94.9)	38 (86.4)	55 (91.7)	27 (96.4)	28 (87.5)
CIRS (total score), median (range)^c^	7 (1–18)	5 (1–15)	7 (2–18)	8.0 (2–20)	7.0 (2–14)	10.5 (4–20)
Body mass index, median (range)^d^	25.0 (18.0–34.3)	25.0 (20.5–33.8)	25.0 (18.034.3)	28.5 (21.5–37.6)	28.5 (21.5–37.6)	28.5 (22.1–37.3)
Exercise, median hours per week (range)	4 (0–20)	4 (0–14)	4 (0–20)	3 (0–15)	2.5 (0–14)	3.5 (0–15)
Smokers, *N* (%)	49 (59.0)	24 (61.5)	25 (56.8)	34 (56.7)	14 (50.0)	20 (62.5)
*N* of pack years for smokers, median (range)	10 (1–50)	5 (1–35)	20 (1–50)	15 (1–80)	12.5 (2–80)	15 (1–60)
Alcohol, median *N* of standard drinks per week (range)^e^	4 (0–70)	4 (0–35)	4 (0–70)	3.5 (0–25)	2 (0–20)	4 (0–25)
Sexually active, *N* (%)	77 (92.8)	37 (94.9)	40 (90.9)	53 (88.3)	27 (96.4)	26 (81.3)
International index of erectile function—five items (IIEF5), median (range)	22 (1–25)	22 (4–25)	21 (1–25)	21 (2–25)	23 (4–25)	18 (2–25)
Erectile dysfunction by IIEF < 22, *N* (%)	41 (49.4)	18 (46.2)	23 (52.3)	36 (60.0%)	13 (46.4)	23 (71.9)
Total testosterone, IA, median (range)^f^	18.0 (15.0–31.0)	18.0 (15.0–31.0)	18.0 (15.0–28.0)	10.6 (6.7–17.8)	12.0 (9.9–14.0)	11.0 (7.3–14.0)
By treatment						
R-CHOP-like regimen	–	–	18.0 (15.0–28.0)	–	–	11.0 (7.3–14.0)
ABVD	–	19.0 (15.0–31.0)	–	–	12.0 (10.0–14.0)	–
BEACOPP	–	17.0 (15.0–27.0)	–	–	12.5 (9.9–14.0)	–

*N*, number; *CIRS*, cumulative illness rating scale; *IA*, immunoassay analysis; *R-CHOP*, rituximab, cyclophosphamide, doxorubicin, vincristine, prednisone; *ABVD*, doxorubicin, bleomycin, vinblastine, dacarbazine; *BEACOPP* bleomycin, etoposide, doxorubicin, cyclophosphamide, vincristine, procarbazine, prednisone.

See earlier published data for full overview of treatment regimens [3]

^a^Four survivors received mixed regimens with unknown number of cycles and number of cycles is therefore not accounted for. However, three had received a combination of ABVD + BEACOPP/BEACOPPe and were placed in the BEACOPP group, and one received ABVD + R-CHOP and was placed in the ABVD group.

^b^All births are stated irrespective of mode of obtained pregnancy: fertility treatment/natural.

^c^Overall score range 0–56.

^d^Calculations: weight in kilograms/height in meters.^2^

^e^Missing data for two survivors, one HL and one DLBCL.

^f^TT concentrations measured at the preliminary study VitalityObs. Testosterone levels stated can differ from cutoff values at inclusion into VitalityCheck because samples were repeated.

The analyses shown in Tables 2 and 3 reflects the androgen status at (1) the time of diagnosis, (2) the time of inclusion in the VitalityObs study (FU1 with TT alone), and (3) the time of inclusion in the VitalityCheck study (FU2 with full androgen status). TT concentrations at follow-up for the 20 survivors with both a diagnosis and a FU2 sample did not differ from the 40 men where TT was not determined prior to lymphoma treatment (mean difference 0.4 nmol/l, 95% CI: − 1.0–1.9), and the 20 men may therefore be considered “representative” of all men in the group of “Low normal TT” at diagnosis (Table 2). Median age was slightly higher for the 20 survivors with “Low normal TT” than for the 30 with “High normal TT,” both at time of diagnosis (median 28.0 and 26.0 years) and follow-up (37.5 and 33.0 years). Despite the difference, the age may be regarded as similar from a biological point of view. In survivors who had cryopreserved semen at time of diagnosis, and therefore had a sample taken at diagnosis, BEACOPP treatment was more common than in survivors who did not cryopreserve semen, most pronounced in survivors with “Low normal TT” at inclusion. There was no difference in treatment intensity between any groups.

**Table 2:** Clinical and epidemiological characteristics of adult male lymphoma survivors according to testosterone group, with subdivisions according to the availability of two sample, at diagnosis and follow-up [3]

	Survivors with “Low normal TT” (extended hormonal assessment) *N* 60					Survivors with “High normal TT” (no extended hormonal analysis) *N* 83				
	20 survivors with two samples (cryopreservation at diagnosis + VitalityCheck)			40 with one sample (VitalityCheck)		30 survivors with two samples (cryopreservation at diagnosis + VitalityObs)			53 with one sample (VitalityObs)	
	Diagnosis	FU2		FU2		Diagnosis	FU1		FU1	
	Median (perc)	Median (perc)	% change (CI)	Median (perc)	Mean diff between FU2 (CI)	Median (perc)	Median (perc)	% change (CI)	Median (perc)	Mean diff between FU1 (CI)
Age, years	28.0 (21.0–36.5)	37.5 (27.9–49.6)	–	55.0 (30.0–66.0)	15.9* (9.5–19.1)	26.0 (19.0–40.5)	33.0 (26.5–48.1)	–	55.0 (31.6–64.4)	16.6* (12.7–20.6)
Follow-up, years	–	9.5 (6.0–14.0)	–	6.5 (4.0–11.0)	1.3* (1.5–4.3)	–	8.0 (4.0–13.0)	–	6.0 (3.0–11.0)	1.3 (− 0.2–2.6)
Treatment, *N* (%)^a^										
ABVD	–	–	–	–	*p* < 0.002*	–	–	–	–	*p* = 0.02*
BEACOPP										
CHOP										
*N* of cycles										
ABVD		6.0	–	4.0	*p* = 0.17		5.0		4.0	*p* = 0.02*
BEACOPP		6.0		6.0			6.0		8.0	
CHOP		6.0		6.0			6.0		6.0	
TT, IA, nmol/l	14.2 (8.1–21.3)	10.6 (8.3–14.6)	21.1%↓* (5.2–34.3)	10.4 (6.8–16.3)	0.4 (− 1.0–1.9)	15.4 (11.3–28.9)	18.0 (15.0–25.6)	22.6%↑* (1.6–47.9)	19.0 (15.0–25.0)	0.4 (− 2.0–1.2)

*N*, number; *FU1*, at VitalityObs; *FU2*, at VitalityCheck; *TT*, total testosterone; *FT*, free testosterone, *SHBG*, sexual hormone binding globulin; *LH*, luteinizing hormone; *CI*, 5–95% confidence interval; *perc*, 5–95% percentiles; *FU1*, follow-up 1; *FU2*, follow-up 2; *HL*, Hodgkin lymphoma; *DLBCL*, diffuse large B-cell lymphoma; *R-CHOP*, rituximab, cyclophosphamide, doxorubicin, vincristine, prednisone; *ABVD*, doxorubicin, bleomycin, vinblastine, dacarbazine; *BEACOPP*, bleomycin, etoposide, doxorubicin, cyclophosphamide, vincristine, procarbazine, prednisone.

See earlier published data for full overview of treatment regimens [3]

^a^All treated with ABVD and BEACOPP were HL survivors; all treated with R-CHOP was DLBCL survivors.

*Statistically significant changes and differences.

**Table 3:** Hormonal analyses of adult male lymphoma survivors according to testosterone group, with subdivisions according to the availability of two sample, at diagnosis and follow-up

	**Survivors with “low normal TT” (extended hormonal assessment)** ***N* 60**					**Survivors with “high normal TT” (no extended hormonal analysis)** ***N* 83**	
	20 survivors with two samples (cryopreservation at diagnosis + VitalityCheck)			40 with one sample (VitalityCheck)		30 survivors with two samples (cryopreservation at diagnosis + VitalityObs)	
	Diagnosis	FU2		FU2		Diagnosis	
	Median (perc)	Median (perc)	% change	Median (perc)	% difference between FU2-20 to FU-40*	Median (perc)	% difference between diagnosis samples
TT, IA, nmol/l	14.2 (8.1–21.3)	10.6 (8.3–14.6)	21.1%↓* (5.2–34.3)	10.4 (6.8–16.3)	5.1%↓ (− 4.7–19.8)	15.4 (11.3–28.9)	10.5% (− 12.6–39.7)
cFT, IA, pmol/l	339 (202–516)	279 (171–368)	21.1%↓* (5.2–34.3)	208 (136–323)	19.1%↓* (4.3–31.3)	350 (196–596)	1.4% (− 20.2–28.9)
SHBG, nmol/l	24.5 (13.9–35.2)	22.2 (15.5–35.4)	4.4%↓ (− 18.2–11.6)	30.3 (18.4–52.5)	34.4%↑* (10.6–63.3)	30.0 (17.8–51.6)	23.4%* (0.4–51.8)
LH, IU/l	5.6 (2.1–9.6)	4.4 (2.0–10.7)	13.4%↓ (− 38.2–21.5)	4.2 (2.3–9.5)	3.9%↑ (− 23.4–40.9)	3.9 (1.9–7.3)	22.7% (− 40.6–0.7)
TT/LH ratio	2.7 (1.2–5.3)	2.8 (1.3–4.5)	11.1%↓ (− 39.8–31.4)	2.3 (1.0–5.2)	10.8%↑ (− 33.1–18.8)	4.4 (1.6) 1.35 (0.46)	42.9%* (3.9–96.7)
cFT/LH ratio	0.06 (0.03–0.16)	0.07 (0.03–0.11)	4.2%↓ (− 41.0–34.2)	0.05 (0.02–0.11)	24.9%↑ (− 45.4–3.3)	0.09 (0.05–0.14)	34.1% (− 2.1–83.5)

*N*, number; *FU1*, at VitalityObs; *FU2*, at VitalityCheck; *TT*, total testosterone; *FT*, free testosterone; *SHBG*, sexual hormone binding globulin; *LH*, luteinizing hormone; *CI*, confidence interval; *FU2*, follow-up 2.

*Significant changes and differences.

In the group of 20 men, in the “Low normal TT” group with both a diagnosis and a follow-up sample, the TT decreased from 14.2 nmol/l at time of diagnosis to 10.6 nmol/l at FU2 corresponding to a decrease of 21.1% (95% CI: 5.2–34.4%) (Table 2). The decrease was not a reflection of change in the concentration of SHBG, and thus cFT also decreased by 21.1% (95% CI: 5.2–34.3) (Table 3). SHBG, LH, TT/LH, and cFT/LH showed non-significant trends toward decreases over time: 4.4% (95% CI: − 18.2–11.6), 13.9% (95% CI: − 38.2–21.5), 11.1% (95% CI: − 39.8–31.4%), and 4.2% (− 41.0–34.2%), respectively. Nine of the 60 survivors (15%) with “Low normal TT” were identified with cFT testosterone concentration below age adjusted reference levels. Of these 5/9 were diagnosed with DLBCL and had received R-CHOP/R-CHOEP, and 4/9 mere diagnosed with HL where three received ABVD and one received BEACOPP. Median TT was comparable to others in the “Low normal TT” group, but cFT was lower (median 190 pmol/l vs 279 pmol/l), SHBG and LH were higher, comorbidity scores were higher, and IIEF5 scores below cutoff for erectile dysfunction (Table 4).

**Table 4:** Androgen status, questionnaire scores, and comorbidity score of nine male survivors of malignant lymphoma with normal TT but low cFT

	cFT	TT	LH	SHBG	IIEF5	CIRS
Mean	186.4	10.0	7.0	35.2	17.1	9
Median (range)	190 (157–216)	10.1 (5.6–14.7)	5.2 (2.4–12.8)	37 (6–54)	17 (2–25)	9 (4–14)

*TT*, total testosterone; *cFT*, calculated free testosterone; *LH*, luteinizing hormone; *SHBG*, sex hormone-binding globulin; *IIEF5*, international index of erectile function questionnaire with 5 questions (scores 0–25); *CIRS*, cumulative illness rating scale.

For the group of “High normal TT,” TT concentrations increased from 15.4 to 18.0 nmol/l corresponding to an increase of 22.6% (95% CI: 1.6–47.9). Medians for the TT/LH ratios and the FT/LH ratios were all lower for survivors with “Low normal TT” (2.8 and 2.3 at follow-up for both subgroups) than for the “High normal TT” (4.4 at follow-up), which is a result of dividing groups according to T concentrations (Table 3). Both TT/LH and FT/LH ratios at the time of diagnosis for the 20 survivors with “Low normal TT” decreased approximately 4% (4.2 and 4.6%). For the group of 40 men with “Low normal TT” but no diagnosis sample, TT was similar to the TT of the 20 men, but cFT was lower, and SHBG concentration higher (34%, 95% CI: 10.6–63.3%) at FU2. No significant differences were seen for the concentrations of LH or the T/LH ratios between the 20 and the 40 men of the group with “Low normal TT.” Thirty survivors with “High normal TT” at FU1 also had a diagnosis sample and differed from the group with “Low normal TT” by having a significantly higher TT at the time of diagnosis and an increase over time of 22.6% (95% CI: 1.6–47.9%) (Table 2), and a higher TT/LH ratio. Furthermore, SHBG concentration in the group of 30 men was comparable to the group of 40 men, and thereby higher than for the group of 20 men (Table 3).

**Fig. 2:**
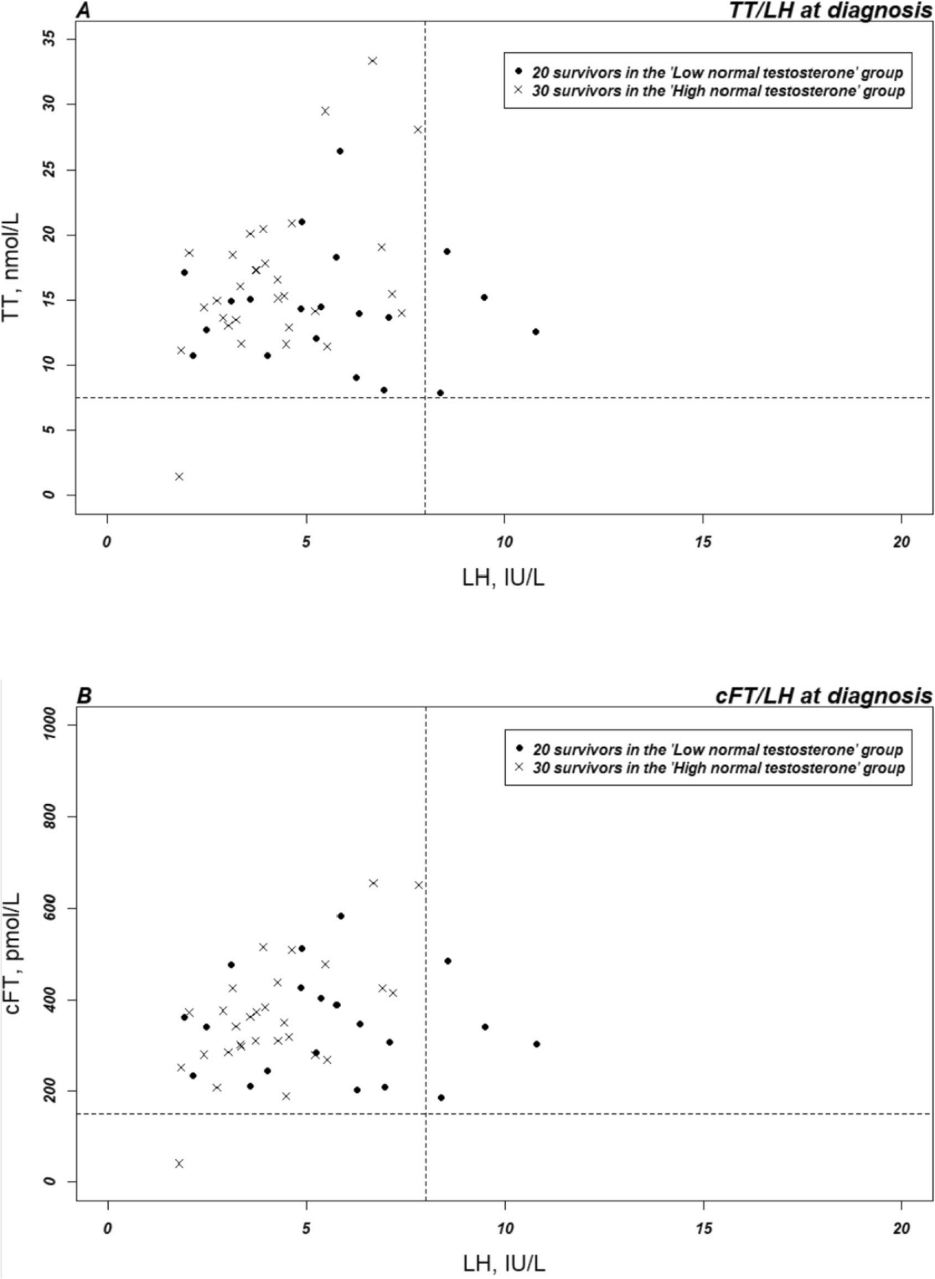

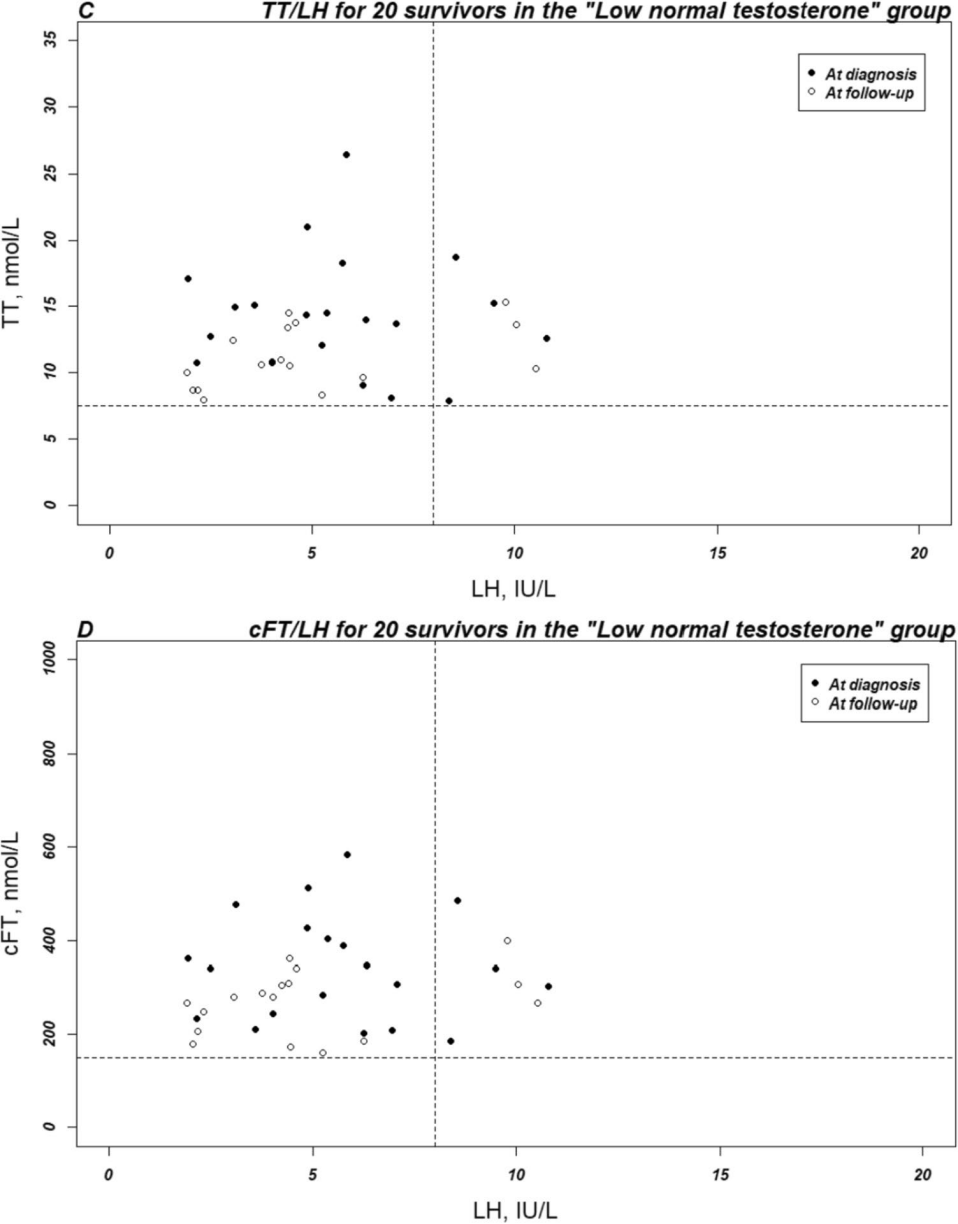
Testosterone-LH ratios for the subgroup of 50 survivors with serum testosterone at both diagnosis and follow-up. TT, total testosterone; cFT, calculated free testosterone; LH, luteinizing hormone. Dashed lines represent reference levels, used at the Department of Growth and Reproduction at Copenhagen University Hospital—Rigshospitalet, for testosterone and luteinizing hormone based on the median age of included survivors (age 45–50 years): TT = 7.5 nmol/l, cFT = 150 pmol/l, LH = 8 IU/l. **A** TT/LH (IA) and **B** FT/LH (IA) at diagnosis the group with “Low normal TT” and “High normal TT.” Survivors with “High normal TT” (black dots) at diagnosis show no sign of compensated TD (no increase in LH), whereas the group with “Low normal TT” (white dots) show signs of compensation with some survivors with LH above reference levels. **C** TT/LH (AI) and **D** FT/LH (IA) at diagnosis and FU2 in the group with “Low normal TT.” For both the TT/LH and the FT/LH ratios, T levels decrease over time. For both time points, some compensation with increased LH is seen

Figure 2 shows that TT/LH and cFT/LH ratios are comparable at diagnosis for all 50 men who had a diagnosis sample, but that ratios for the 20 men, in the “Low normal TT” group with both a diagnosis and a follow-up sample, decreased over time, because of decreased T concentration combined with stable LH concentration, indicating some degree of downregulation of the pituitary gonadotropin secretion.

Analyses included in this study were performed with either IA or LC–MS/MS. IA was used for the analyses performed in all three parts of this assessment (at diagnosis, at FU1, and FU2) and LC–MS/MS was performed on FU2 alone. Differences between the analyses were tested, and IA was found comparable between FU1 and FU2. Furthermore, LC–MS/MS and IA were found comparable on Bland–Altman plots (supplementary data). Only IA results are therefore stated in this article for simplicity.

## Discussion

The two main findings of this study are (1) that men treated with BEACOPP are at increased risk of decreasing TT and (2) that screening by use of TT alone is insensitive in diagnosing men with biologically low testosterone. Surprisingly, TT was comparable between younger HL survivors and older DLBCL survivors, despite the expectation of older men having lower Leydig cell function than younger men. One difference between the two age groups was the difference in treatment regimen. The younger HL survivors were more often treated with harsh chemotherapy—BEACOPP. This indicates that even younger survivors of malignant lymphoma, who receive treatment with BEACOPP, should be assessed endocrinologically because of an increased risk of lower Leydig cell function. Routinely, cryopreservation is offered to younger patients with HL who are expected to receive more toxic chemotherapy regimens, meaning that the low age of the subgroup of 20 survivors from the “Low normal TT” group might be expected. We have previously described BEACOPP-treated survivors with a trend toward worse erectile function [3] and others have found BEACOPP-treated survivors to have worse sexual health than ABVD-treated and/or controls [20, 21]. Behringer et al. found male lymphoma survivors with impaired fertility and testosterone deficiency in up to 28% of BEACOPP-treated [22]. Furthermore, the toxic effect of BEACOPP on gonadal function, with impaired fertility as a consequence, is a well-established late effect [23]. In the current study, a trend toward more severe gonadal damage, as evidenced by lower testosterone levels, was also found. BEACOPP must be regarded as a high-risk treatment for gonadal function as well as sexual health of male malignant lymphoma patients. The differential decrease in TT for the two groups of survivors with “High normal TT” and “Low normal TT” at inclusion into the current study supports the hypothesis that BEACOPP-treated survivors constitute a high-risk group. Furthermore, decreasing testosterone concentrations with no compensatory increase in LH over time could indicate some level of downregulation of pituitary secretion of LH, perhaps an effect by treatment with BEACOPP, even though evidence from previous research is not compelling [24].

In the era of new targeted therapy, patterns of side effects will undoubtedly change. This can also have an impact on the occurrence of gonadal toxicity. In DLBCL, the combination of the antibody–drug conjugate polatuzumab targeting CD79b combined with R-CHP has shown promising results in the POLARIX study [25]. Bispecific monoclonal antibodies are also tested first line in early clinical trials. In Hodgkin lymphoma, a phase III study tested the antibody–drug conjugate brentuximab vedotin plus AVD versus ABVD [26], and another large phase III study is underway investigating the brentuximab vedotin regimen BrECADD (brentuximab vedotin, etoposide, doxorubicin, cyclophosphamide, dacarbazine, dexamethasone) versus BEACOPP [27]. Both therapies have been approved in several countries for first-line therapy, and it is expected that these regimens will be more common in the near future. Despite a changed side effect profile, many new biologically targeted treatments also affect gonadal function and both polatuzumab and brentuximab are accompanied by neurotoxicity and immune-related adverse reactions in a substantial proportion of patients. Research investigating these adverse gonadal reactions are scarce, and further focus on both spermatogenesis and Leydig cell function ought to be initiated. In the present study, we found a comparable decrease in TT and cFT over time for the 20 men in the “Low normal TT” group. For TT concentrations to be mirrored by cFT, SHBG has to be unaltered after chemotherapy. We found SHBG to be slightly non-significantly decreased at follow-up, but higher in the older survivors constituting the 40 men of the “Low normal TT” group. This is in line with SHBG being known to increase by increasing age [6]. Despite the increase, cFT was within reference levels for 85% of the “Low normal TT” group, where nine survivors (15%) had cFT below age adjusted reference levels in combination with normal TT indicating that some survivors with TD goes undiagnosed with TT screening alone. In the general population (40–79 years of age), the prevalence has been shown to be 8.3% [5], and normal TT with cFT below reference level is therefore more common in our cohort of male lymphoma survivors. This indicates that using TT alone for the screening of testosterone deficiency in the initial study VitalityObs was not fully sufficient to detect TD and would have increased the initial prevalence of TD from 4.7 to 9.9% in the VitalityObs study. A previous study has also shown an independent relationship between the TT and the cFT [4]. The nine survivors are not visible as below reference levels in Fig. 2, because the reference levels are shown for the median age of the included survivors. This was accepted because this figure was meant to show the overall course of the T/LH ratios.

Many subgroups were identified in this study, and it is important to clarify whether results from the small subgroup of 20 are representative of the full “Low normal TT” group, and whether the 60 survivors included in the VitalityCheck study are representative of the 172 survivors included in the VitalityObs study. The 20 survivors with hormonal evaluation at both diagnosis and FU2 differed from the 40, but combined the 60 survivors are considered representative of the 172 survivors of the initial study, because gonadal function in this group was the most impaired. Therefore, the identification of younger BEACOPP-treated survivors and older survivors as high-risk groups is deemed appropriate. Twenty-one survivors were not included in the “Low normal TT” group in the VitalityCheck study but were comparable to the group of 40 men without a diagnosis sample, where 15/21 would have been characterized as such (data not shown). Including these 21 men would probably not have changed results.

Some limitations of the current study design exist. Sample sizes were small, and our study is based on collection of samples from archives combined with samples taken at two different times of follow-up. Analyses compared have therefore been performed on different analysis machines (between archive samples, VitalityObs samples and VitalityCheck samples), and at different laboratories (VitalityObs performed at different laboratory), which could impact reliability of the results. Furthermore, sampling was not consistently performed in the morning for all archived samples, which could result in affected testosterone concentrations being less comparable to those sampled in the morning, because of diurnal changes in TT.

We investigated the differences between TT measured using immunoassays at FU1 and 2 and found them comparable. However, TT measured at FU2 was consistently 2 nmol/l higher than for samples taken at FU1. Because of this difference, we did not compare these directly. However, if taking this difference into account, the difference between FU1 in the “High normal TT” group and FU2 in the “Low normal TT” groups would only be even more pronounced. Furthermore, if mean TT at diagnosis in the “Low normal TT” group was 2 nmol/l higher than the stated, the difference between the diagnosis and the FU2 value would also be more pronounced. Therefore, we did not consider this difference to be a problem. However, a prospective design would provide more stringent groups of equal numbers, and a proper control group would be preferable.

We have omitted LC–MS from these analyses, because validation of our results found the IA analyses and the LC–MS analyses to yield the same results (online source). Because we have only IA analyses for comparison between diagnosis and follow-up, we chose to focus on these analyses. However, LC–MS is considered the gold standard, meaning future prospective studies should include LC–MS alongside IA samples. IA is still relevant in research studies because it is used frequently in the clinical endocrinologic evaluation of patients and survivors.

For the calculation of cFT, the method proposed by Vermeulen et al. was used [19], which is considered to be a precise method. However, because we did not have albumin analyses available, the albumin value was fixed for the calculations. Earlier, we hypothesized that liver damage could result in altered SHBG concentrations, and if this is correct albumin concentrations could be hypothesized to be altered as well. However, in this study, we did not find SHBG levels to be above reference levels, making this of less concern.

When investigating pre-treatment testosterone, it would be optimal to collect samples before the onset of disease in a large cohort study investigating gonadal health over time in the general population, and not at diagnosis of a lymphoma disease. In times of disease, gonadal function can be impaired, reducing production of testosterone. In this study, pre-treatment testosterone concentrations could therefore be somewhat reduced [28, 29], and the true effect of treatment even greater.

## Conclusion

When using TT as screening, the presence of biologically relevant testosterone deficiency in at least 5.2% of lymphoma survivors may be missed. Thus, screening of older malignant lymphoma survivors and survivors treated with harsh combination chemotherapy, BEACOPP, should rely on cFT. Especially men treated with BEACOPP appear to be of increased risk of TD and should be followed more closely during follow-up. Prospective studies with longitudinal follow-up of patients at high risk of TD are needed.

### Supplementary Information

Below is the link to the electronic supplementary material.

Supplementary file1 (DOCX 337 KB)

## Data Availability

Data is not available because of patient sensitive data.

## References

[1] Bersvendsen HS (2020). Sexual function in long-term male lymphoma survivors after high-dose therapy with autologous stem-cell transplantation. Bone Marrow Transplant.

[2] Tsatsou I (2023). Sexual function of male survivors of hematological malignancy treated by autologous hematopoietic stem cell transplantation: a multicenter controlled observational study. J Sex Marital Ther.

[3] Micas Pedersen S (2023). Sexual dysfunction is highly prevalent in male survivors of malignant lymphoma. Sex Med.

[4] Narinx N (2022). Role of sex hormone-binding globulin in the free hormone hypothesis and the relevance of free testosterone in androgen physiology. Cell Mol Life Sci.

[5] Antonio L (2016). Low free testosterone is associated with hypogonadal signs and symptoms in men with normal total testosterone. J Clin Endocrinol Metab.

[6] Andersson AM, Jensen TK, Juul A, Petersen JH, Jørgensen T, Skakkebæk NE (2007). Secular decline in male testosterone and sex hormone binding globulin serum levels in Danish population surveys. J Clin Endocrinol Metab.

[7] Wittert G, Grossmann M (2022). Obesity, type 2 diabetes, and testosterone in ageing men. Rev Endocr Metab Disord.

[8] Dandona P, Dhindsa S, Chaudhuri A, Bhatia V, Topiwala S (2008). Hypogonadotrophic hypogonadism in type 2 diabetes, obesity and the metabolic syndrome. Aging Male.

[9] Kaufman JM (2022). Diagnosis of hypogonadism in ageing men. Rev Endocr Metab Disord.

[10] Howell SJ, Radford JA, Ryder WDJ, Shalet SM (1999). Testicular function after cytotoxic chemotherapy: evidence of Leydig cell insufficiency. J Clin Oncol.

[11] Kauppila M (1998). The hypothalamus-pituitary-gonad axis and testicular function in male patients after treatment for haematological malignancies. J Intern Med.

[12] Tsatsoulis A, Whitehead E, John ST, Shalet SM, Robertson WR (1987). The pituitary-Leydig cell axis in men with severe damage to the germinal epithelium. Clin Endocrinol (Oxf).

[13] Tsatsoulis A, Shalet SM, Robertson WR, Morris ID, Burger HG, DE Kretser DM (1988). Plasma inhibin levels in men with chemotherapy-induced severe damage to the seminiferous epithelium. Clin Endocrinol (Oxf).

[14] Anderson LJ (2022). Androgens and estrogens predict sexual function after autologous hematopoietic stem cell transplant in men. Andrology.

[15] Clark ST, Radford JA, Crowther D, Swindell R, Shalet SM (1995). Gonadal function following chemotherapy for Hodgkin’s disease: a comparative study of MVPP and a seven-drug hybrid regimen. J Clin Oncol.

[16] Waxman J (1985). The gonadal effects of cancer and its treatment. Clinics in Oncology.

[17] Greenfield DM (2007). Prevalence and consequences of androgen deficiency in young male cancer survivors in a controlled cross-sectional study. J Clin Endocrinol Metab.

[18] Arboe B (2016). The Danish National Lymphoma Registry. Clin Epidemiol.

[19] Vermeulen A, Verdonck L, Kaufman JM (1999). A critical evaluation of simple methods for the estimation of free testosterone in serum. J Clin Endocrinol Metab.

[20] Behringer K (2013). Sexual quality of life in Hodgkin Lymphoma: a longitudinal analysis by the German Hodgkin Study Group. Br J Cancer.

[21] Eeltink CM (2020). Self-reported sexual function in sexually active male Hodgkin lymphoma survivors. Sex Med.

[22] Behringer K (2013). Gonadal function and fertility in survivors after Hodgkin lymphoma treatment within the German Hodgkin study group HD13 to HD15 trials. J Clin Oncol.

[23] Amin MSA (2021). ABVD and BEACOPP regimens’ effects on fertility in young males with Hodgkin lymphoma. Clin Transl Oncol.

[24] Müller J (2003). Impact of cancer therapy on the reproductive axis. Horm Res.

[25] Tilly H (2022). Polatuzumab vedotin in previously untreated diffuse large B-cell lymphoma. N Engl J Med.

[26] Connors JM (2018). Brentuximab vedotin with chemotherapy for stage III or IV Hodgkin’s lymphoma. N Engl J Med.

[27] P. Borchmann et al (2022) Treatment related morbidity in patients with classical hodgkin lymphoma: Results of the ongoing, randomized phase III HD21 trial by the German Hodgkin study group. Blood (2022) 140 (Supplement 1):771–773. 10.1182/blood-2022-165917

[28] Vigersky RA, Chapman RM, Berenberg J, Glass AR (1982). Testicular dysfunction in untreated Hodgkin’s disease. Am J Med.

[29] Wigny KMGJ (2016). Gonadal function in boys with newly diagnosed cancer before the start of treatment. Hum Reprod.

[30] Harris PA (2019). The REDCap consortium: building an international community of software platform partners. J Biomed Inform.

[31] Harris PA, Taylor R, Thielke R, Payne J, Gonzalez N, Conde JG (2009). Research electronic data capture (REDCap)-a metadata-driven methodology and workflow process for providing translational research informatics support. J Biomed Inform.

