# Supplementary material for: Men treated with BEACOPP for Hodgkin lymphoma may be at increased risk of testosterone deficiency
Source: Ann Hematol. 2023 Oct 23;103(1):227–39. doi: 10.1007/s00277-023-05512-y (PMC10761514; doi:10.1007/s00277-023-05512-y)
Supplement: Supplementary file 1 — Supplementary file1 (DOCX 337 KB) [file 277_2023_5512_MOESM1_ESM.docx]

**Online sources**

**Men treated with BEACOPP for Hodgkin Lymphoma may be at increased risk of testosterone deficiency**

Authors: Signe Micas Pedersen, Claus Larsen Feltoft, Torsten Holm Nielsen, Peter Brown, Anne Ortved Gang, Lars Møller Pedersen, Niels Jørgensen

**Information on the 21 excluded survivors**

|  | The 21 survivors not included in *VitalityCheck* | | The 60 survivors included in *VitalityCheck* |
| --- | --- | --- | --- |
|  | Mean (SD) | median | Median (range) |
| Age at FU1 | 49.9 (11.4) | 53 | 47 (27-67) |
| Age at diagnosis | 43.1 (12.3) | 47 | 40 (19-61) |
| Follow-up | 6.7 (2.4) | 6.0 | 8.0 (4.0-14.0) |
| Chemotherapy regimen  -ABVD  -BEACOPP  -RCHOP  -other | 11 (52.4)  2 (9.5)  8 (38.1) |  | 17 (30.4)  7 (12.5)  31 (55.4)  1 (1.8) |

**Supplementary Figure A –** The comparability of the immunoassay analyses performed at inclusion into the VitalityObs and immunoassay analyses performed at inclusion into the VitalityCheck study


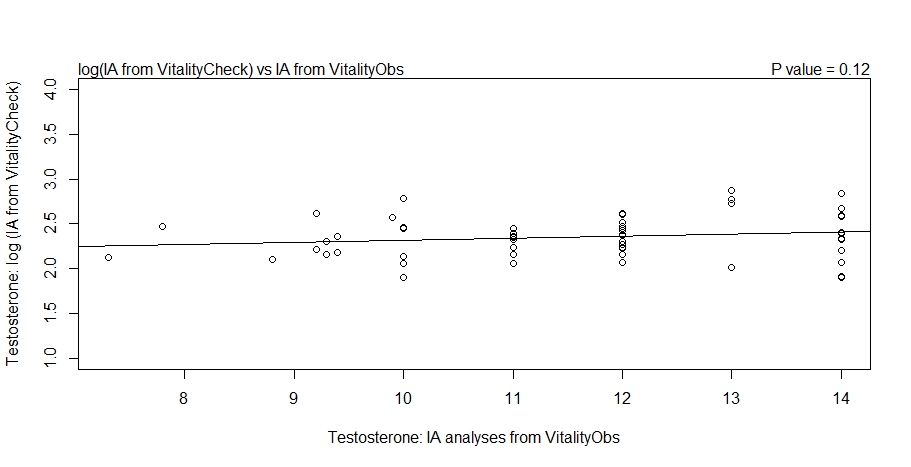


A constant difference between the immunoassay analyses is seen, with analyses performed at inclusion into the Vitality Check study being consistently 2.1 units higher than at inclusion into the Vitality Obs study. Analyses are performed at two different laboratories with approximately one year in between.

**Supplementary Figure B** – Bland-Altman plot of the difference between testosterone analysis performed on immunoassays compared to liquid chromatography mass spectrometry assays for 60 male survivors of malignant lymphoma


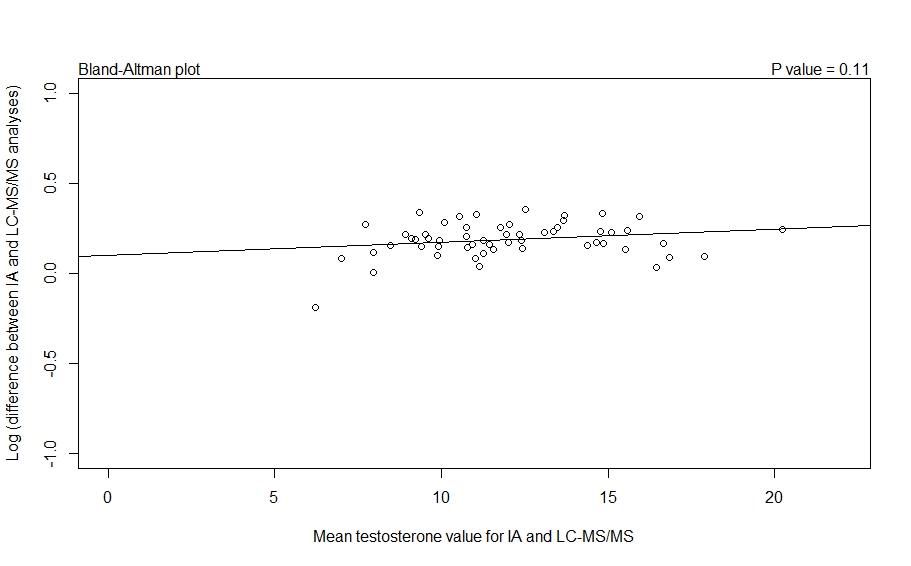


Bland-Altman plot of the natural logarithm to differences between the means for LC-MS analyses and immunoassay analysis (dependent variable) as a function of the raw mean of total testosterone measurements ((LC-MC – immunoassay)/2) (independent variable). No significant difference between the two analysis methods were seen. However, LC-MS values were systematically but non-significantly 0,1 units higher than immunoassay values (95% confidence interval: -0.0056 – 0.2099).

**Supplementary for Fig. 2-** Testosterone-LH ratios for the subgroup of 50 survivors with serum testosterone at both diagnosis and follow-up.


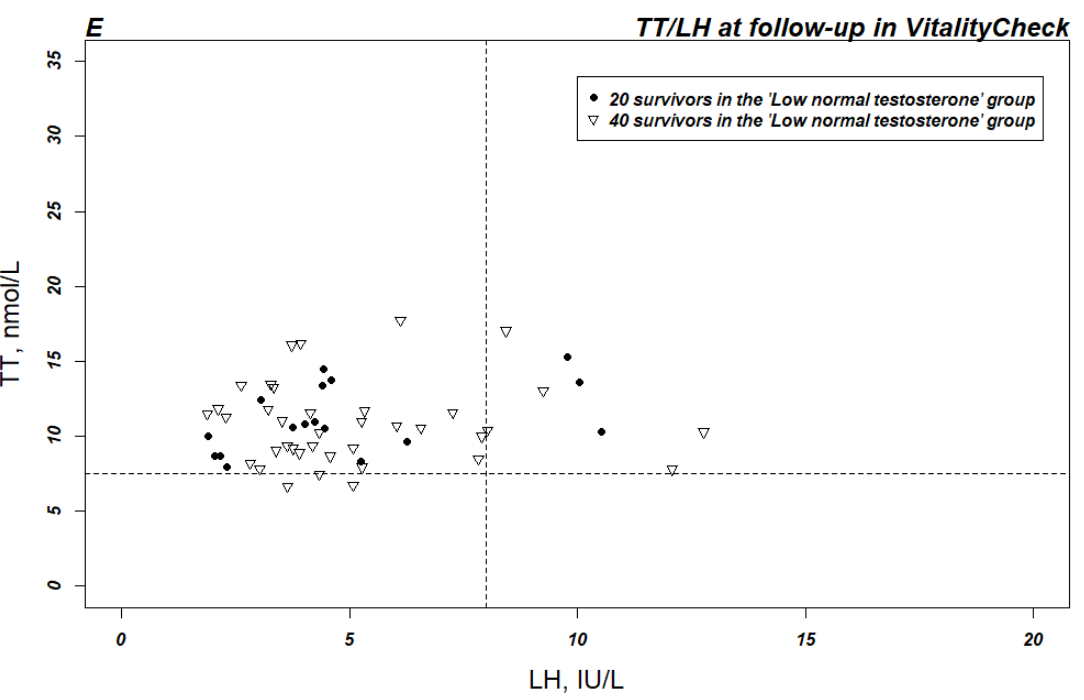

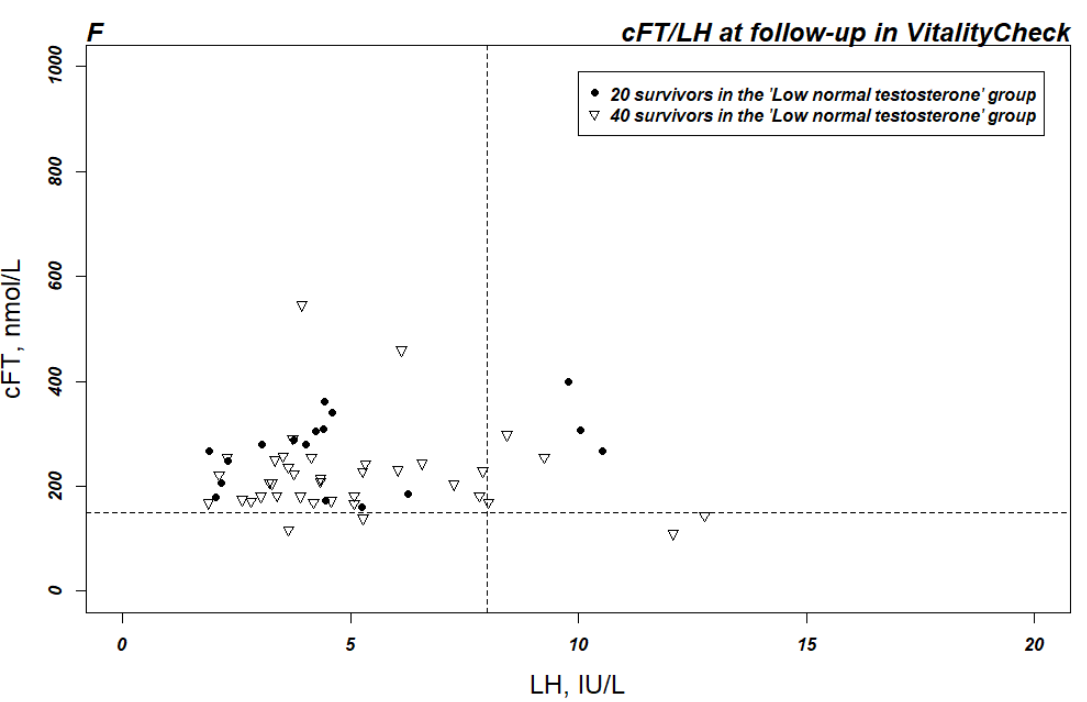


E: TT/LH and F: FT/LH at follow-up for the group with “Low normal TT” according to whether they had a blood sample at diagnosis

Both TT/LH and FT/LH ratios were similar in the two groups, with signs of some degree of compensation.
